# Can we have our steak and eat it: The impact of breeding for lowered environmental impact on yield and meat quality in sheep

**DOI:** 10.3389/fgene.2022.911355

**Published:** 2022-09-16

**Authors:** S. J. Rowe, S. M. Hickey, W. E. Bain, G. J. Greer, P. L. Johnson, S. Elmes, C. S. Pinares-Patiño, E. A. Young, K. G. Dodds, K. Knowler, N. K. Pickering, A. Jonker, J. C. McEwan

**Affiliations:** ^1^ Invermay Agricultural Centre, AgResearch Ltd., Mosgiel, New Zealand; ^2^ Ruakura Research Centre, AgResearch Ltd., Hamilton, New Zealand; ^3^ Grasslands Research Centre, AgResearch Ltd., Palmerston North, New Zealand

**Keywords:** sheep, respiration chamber, breeding, genetic correlation, methane emissions, meat quality

## Abstract

Global agreements in place to reduce methane emissions in livestock are a potential threat to food security. Successful but independent breeding strategies for improved production and lower methane are in place. The unanswered questions are whether these strategies can be combined and how they impact one another, physically and economically. The New Zealand economy is largely dependent on pastoral agriculture from grazing ruminants. The sheep industry produces ∼20 million lamb carcasses for export each year primarily from grass. Methane emitted from the fermentation of forage by grazing ruminants accounts for one-third of all New Zealand’s greenhouse gas emissions. Here, we use sheep selection lines bred for divergent methane production and large numbers of their relatives to determine the genetic and phenotypic correlations between enteric methane emissions, carcass yield, and meat quality. The primary objectives were to determine whether previously shown physiological differences between methane selection lines (differing by ∼12% in methane) result in a negative impact on meat production and quality by measuring close relatives. The results show no negative effects of breeding for lowered methane on meat and carcass quality. Gross methane emissions were highly correlated with liveweight and measures of carcass weight and negatively correlated with dressing-out percentage and fat yield (GR). Trends were similar but not significant for methane yield (g CH_4_/kg DMI). Preliminary evidence, to date, shows that breeding for low methane may result in animals with higher lean yields that are economically favorable even before carbon costs and environmental benefits are taken into account. These benefits were seen in animals measured for methane on fixed intakes and require validation on intakes that are allowed to vary.

## Introduction

**FIGURE 1:**
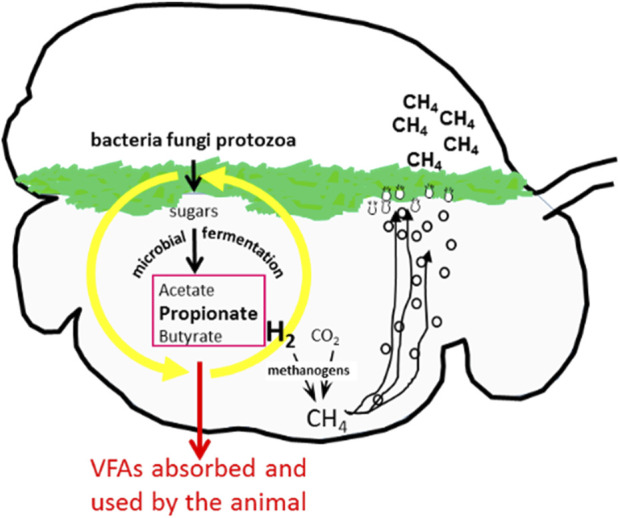
Complex polymers such as cellulose are partially digested in the ruminant foregut or rumen. During the fermentation process volatile fatty acids are released and methanogens metabolise hydrogen to form methane. Image courtesy of Graeme Attwood, AgResearch Ltd.

Greater than one-quarter of the Earth’s total land mass is used for grazing ruminants (FAO, 2006). The evolutionary adaptation of the ruminant to convert pasture to animal products such as meat, milk, and fibre may have been successful, but ruminant production has an unwanted by-product that is highly detrimental to the environment. During the breakdown and fermentation of plant material in the rumen by symbiotic microbes, hydrogen is produced (Figure 1). This hydrogen is largely utilized by rumen methanogens and eructed by the animal as methane, a potent greenhouse gas in terms of global warming potential. The sustainability of profitable livestock farming throughout the world is increasingly being threatened by methane emissions. Enteric methane from grazing ruminants is the source of 35% of New Zealand’s total greenhouse gas emissions and, globally, livestock contributes approximately 14–18% of the total greenhouse gas emissions (FAO, 2006; Ministry for the Environment, 2021). Methane is not only an environmentally detrimental waste product but also represents ∼2–10% energy loss to the animal (Goopy et al., 2013). Methane emissions per unit of dry matter intake (i.e., methane yield) have been shown to vary between individual sheep, and furthermore, these differences are heritable (Pinares-Patiño et al., 2013; Goopy et al., 2013; Jonker et al., 2018). One strategy to mitigate methane emissions is therefore to select breeding stock for lowered emissions (Clark, 2009). In New Zealand, low and high methane yield selection lines were established through screening research flocks for sheep that were extreme for methane yield. Then, the methane yield selection line flocks were closed to outside animals, and selection was within each line (Pinares-Patiño et al., 2013; Jonker et al., 2018; Rowe et al., 2019). After 10 years of selection, the high and low methane yield lines have an ∼12% difference in methane yield (Rowe et al., 2019).

Before undertaking the use of breeding for adaptation strategies in ruminants such as low methane emissions, it will be crucial for us to understand the impact of this on the animal’s digestive physiology and any potential negative outcomes on animal production (Waghorn and Hegarty, 2011).

Evidence for differences in particle retention time, digesta passage rate, and rumen size in sheep bred for low methane (Goopy et al., 2013; Bain et al., 2014; Waite et al., 2019) suggests that there are associated physiological changes in the alimentary tract when breeding for methane. There is also evidence for different gut microbiota leading to different fermentation profiles, ultimately delivering a different source of energy to the animal (Hess et al., 2020; Bilton et al., 2021). These changes might affect the energy metabolism and partitioning in the animal (Bergman, 1990) with potential effects on carcass characteristics.

We hypothesize that the physiological differences associated with low-methane-yield sheep would have the potential to affect the growth and performance of the sheep and the subsequent quality and conformation of the carcass. The objectives of the current study were to determine the relationship between methane emissions and carcass, and meat quality characteristics.

## Materials and methods

All animal experiments were conducted to meet the guidelines of the 1999 New Zealand Animal Welfare Act and AgResearch Code of Ethical Conduct and were approved by the AgResearch Grasslands (Palmerston North, NZ) and AgResearch Invermay (Mosgiel, NZ) animal ethics committees.

### Animals

Trait data from selected New Zealand sheep flocks, born between 2002 and 2013, were obtained from animals recorded on the Sheep Improvement Limited (SIL) database (Newman et al., 2009). Methane data were obtained from the methane yield selection lines (SIL Flock ID 3633, flock 1). In brief, the development of the methane yield selection lines was based on the screening of progeny from a research flock (SIL Flock ID 2638, flock 2; Jonker et al., 2018) and three New Zealand central progeny test (CPT) flocks (SIL Flock IDs 4640, 4757, and 9153, flocks 3, 4, and 5, respectively; McLean et al., 2006) for methane yield (g CH_4_/kg DMI) as detailed by Pinares-Patino et al. (2013) and Jonker et al. (2018). The lines were created from the progeny of the top and bottom 10 sires. The lines were closed in 2012, and all sires used from 2012 onward were born in the methane yield selection flock. The lines are currently maintained at 100 ewes per line. In this study, 1,825 animals born in 2007 and 2009–13 provide methane yields for correlation with carcass data collected from over 25,000 animals in the 5 flocks mentioned earlier. All animals were born and managed in a ryegrass-based pastoral grazing system.

### Measurements

A summary of the traits and their abbreviations are provided in Table 1. The number of records for each methane, ultrasound, live animal, and carcass trait is provided in Table 2. For the meat and carcass traits, a record refers to a single animal, whereas for methane traits, the number of records reflects multiple measures on each animal. Supplementary Appendix Table SA1 lists the traits and birth flocks, and the birth years from which the data were sourced.

**TABLE 1:** Methane, live animal, ultrasound, carcass, and meat quality trait description with details of final mixed model effects and covariates for genetic parameter analysis.

Trait	Abbreviation	Contemporary group^a^	Fixed effects^a^	Covariates^a^
Methane
Gross methane, g/d	CH_4_, g/d	cg4, cg5	brr	bdev
Methane yield, g/kg DMI	CH_4_/DMI	cg4, cg5	—	—
Live animal
Liveweight (aged 8 months), kg	LWt	flk.cgx.sex	brr, aod	bdev
Preslaughter weight, kg	PRESLT	flk.cgx.sex.PreSltMob	brr, aod	SltrAgeDev
Ultrasound fat depth, mm	FDM	flk.cgx.sex	brr, aod	bdev, PRESLT
Ultrasound eye muscle^b^ depth, mm	EMD	flk.cgx.sex	brr, aod	bdev, PRESLT
Ultrasound eye muscle^b^ width, mm	EMW	flk.cgx.sex	brr, aod	bdev, PRESLT
Carcass
Carcass weight^c^, kg	Carc Wt	CGXext	brr, aod	SltrAgeDev
Carc Wt/PRESLT, %	DO%	CGXext	brr, aod	SltrAgeDev, Carc Wt
GR, mm	CGRM	CGXext	brr, aod	SltrAgeDev, Carc Wt
Carcass length, cm	CLGTH	CGXext	brr, aod	SltrAgeDev, Carc Wt
Leg length, cm	LEGLGTH	CGXext	brr, aod	SltrAgeDev, Carc Wt
Butt circumference, cm	CBUTT	CGXext	brr, aod	SltrAgeDev, Carc Wt
Eye muscle area^b^, cm	CEMA	CGXext	brr, aod	SltrAgeDev, Carc Wt
VIAscan carcass weight, kg	VSCWT	CGXext. VSCWTm	brr, aod	SltrAgeDev
VIAscan GR, mm	VSGR	CGXext. VSCWTm	brr, aod	SltrAgeDev, VSCWT
VIAscan leg lean yield, %	VSLEG	CGXext. VSCWTm	brr, aod	SltrAgeDev, VSCWT
VIAscan loin lean yield, %	VSLOIN	CGXext. VSCWTm	brr, aod	SltrAgeDev, VSCWT
VIAscan shoulder lean yield, %	VSSHLD	CGXext. VSCWTm	brr, aod	SltrAgeDev, VSCWT
VIAscan total lean yield, %	VSYLD	CGXext. VSCWTm	brr, aod	SltrAgeDev, VSCWT
Meat quality
Carcass pH	CPH	CGXext	brr, aod	SltrAgeDev, Carc Wt
Loin pH	LPH	CGXext. SHFm	brr, aod	SltrAgeDev, Carc Wt
Carcass fat color L*	FATL	CGXext	brr, aod	SltrAgeDev, Carc Wt, CPH
Carcass fat color a*	FATA	CGXext	brr, aod	SltrAgeDev, Carc Wt, CPH
Carcass fat color b*	FATB	CGXext	brr, aod	SltrAgeDev, Carc Wt, CPH
Carcass loin color L*	LOINL	CGXext	brr, aod	SltrAgeDev, Carc Wt, CPH
Carcass loin color a*	LOINA	CGXext	brr, aod	SltrAgeDev, Carc Wt, CPH
Carcass loin color b*	LOINB	CGXext	brr, aod	SltrAgeDev, Carc Wt, CPH
Marbling score^d^	MARB	CGXext. SHFm	brr, aod	SltrAgeDev, Carc Wt
Tenderness, shear force, kgF	SHF	CGXext. SHFm	brr, aod	SltrAgeDev, pHdev, pHdev^2^, Carc Wt

a brr: birth rearing rank; bdev: birth day deviation; aod, age of dam; cg4: birth year.birth flock.sex; cg5: recording year.lot (mob of 96 animals).group (sub-mob of up to 24 animals within a lot, measured contemporaneously).round (measurement time 14 days apart); SltrAgeDev: slaughter age deviation; pHdev: pH deviation; cgx: SIL numbered (year.weaning mob.bdev group.LW6/8 mob); CGXExt: birth flock.sex.cgx.pre-slaughter mob.slaughter mob, where “.” indicates an interaction and its lower order terms.

b Eye muscle is the colloquial name for the M. longissimus.

c Carcass weight represents either hot carcass weight, cold carcass weight, or VIAscan® carcass weight.

d Marbling is measured using a subjective score, where 1 = no marbling through 5 = high levels of marbling.

**TABLE 2:** Number of records/animals, mean, phenotypic standard deviation, and heritability estimate for methane, live animal, ultrasound, carcass, and meat quality traits.

Trait	No.	Mean	σ_p_	h^2^ (s.e.)
Methane				
Gross methane, g/d	7,722	24.3	3.05	0.25 (0.04)
Methane yield, g/kg DMI	7,721	15.4	1.5	0.11 (0.02)
Live animal				
Liveweight (aged 8 months), kg	19,437	42.3	4.66	0.52 (0.02)
Preslaughter weight, kg	17,583	41.3	3.45	0.25 (0.02)
Ultrasound fat depth, mm	15,230	2.90	1.07	0.43 (0.03)
Ultrasound eye muscle depth, mm	14,358	25.6	2.24	0.55 (0.02)
Ultrasound eye muscle width, mm	12,215	62.7	4.40	0.45 (0.03)
Carcass				
Carcass weight, kg	18,288	17.9	1.91	0.42 (0.02)
Carc Wt/PRESLT, %	15,943	43.2	1.72	0.40 (0.03)
GR, mm	10,529	6.13	2.03	0.55 (0.03)
Carcass length, cm	9,230	84.4	1.96	0.42 (0.03)
Leg length, cm	9,222	27.4	1.26	0.50 (0.03)
Butt circumference, cm	7,493	64.2	1.41	0.33 (0.03)
Eye muscle area, cm	7,965	11.8	1.28	0.65 (0.04)
VIAscan carcass weight, kg	17,831	18.1	1.9	0.42 (0.03)
VIAscan GR, mm	11,843	5.82	2.22	0.52 (0.03)
VIAscan leg lean yield, %	17,499	22.2	1.14	0.45 (0.02)
VIAscan loin lean yield, %	17,499	14.5	0.72	0.33 (0.02)
VIAscan shoulder lean yield, %	17,499	17.6	0.83	0.49 (0.02)
VIAscan total lean yield, %	17,499	54.1	2.25	0.50 (0.02)
Meat quality				
Carcass pH	6,950	5.7	0.14	0.14 (0.03)
Loin pH	4,861	5.8	0.13	0.24 (0.04)
Carcass fat color L*	6,928	71	3.03	0.20 (0.03)
Carcass fat color a*	6,927	6.31	2.33	0.23 (0.03)
Carcass fat color b*	6,928	11.2	2.42	0.27 (0.03)
Carcass loin color L*	6,880	37.3	1.78	0.22 (0.03)
Carcass loin color a*	6878	18.5	1.27	0.19 (0.03)
Carcass loin color b*	6,873	8.37	0.89	0.14 (0.03)
Marbling score	3,159	2.77	0.6	0.31 (0.05)
Tenderness, shear force, kgF	4,853	6.52	1.88	0.37 (0.05)

#### Methane

**FIGURE 2:**
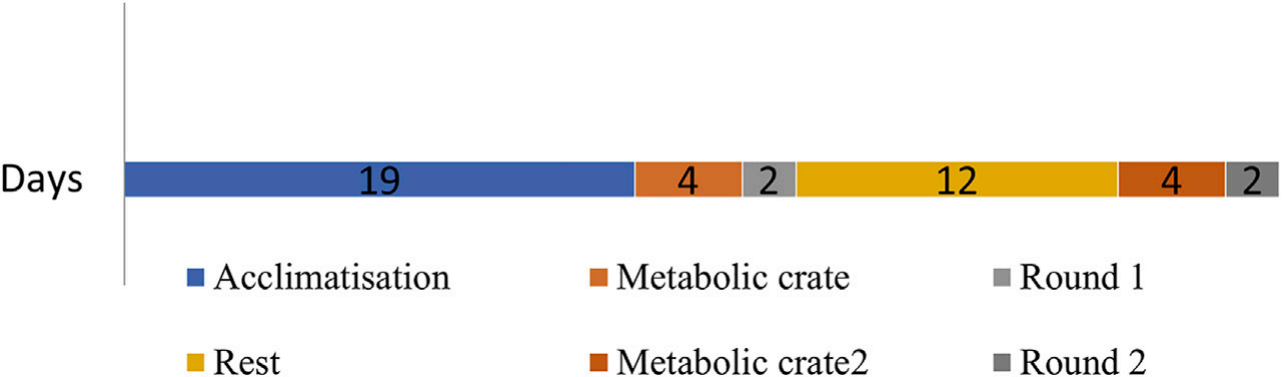
A typical timeline for two rounds of methane measurements. Numbers indicate days. Acclimatisation involved housing animals on a Lucerne pellet diet. Round 1 and Round 2 are methane measurements carried out in respiration chambers.

Traits recorded were gross emissions (CH_4_, g/d) and methane yield (g CH_4_/kg DMI). Enteric methane emissions were measured on male and female lambs between 5 and 10 months of age (30–40 kg liveweight) in a facility with 24 respiration chambers as described by Pinares-Patino et al. (2013) and Jonker et al. (2018). The animals were acclimatized in pens for 19–21 days to a lucerne pellet diet. This was followed by two measurement rounds (R1 and R2) of 48 h in the respiration chambers, with the rounds separated by a 10- to 15-day interval (Figure 2). In each round, individual feed dry matter intake (DMI) was measured in metabolic crates (2 days) and then in respiration chambers (2 days). The feeding level was based on the liveweight and was 2.0 times the estimated maintenance metabolizable energy requirements (CSIRO, 1990). Animals were measured in batches of 96. Both in R1 and R2, individuals were randomly allocated to measurement groups (4 groups) and 1 of 24 respiration chambers, and typically, 10 progeny per sire were randomly selected to be measured for gross emissions and methane yield. Each methane record represents 24-h continuous monitoring in the respiration chambers. There were 7,722 methane records collected from 1,825 animals (344 males and 1,481 females). The methane measurement trials with CH_4_ yield selection line progeny in three birth years have confirmed that the methane phenotype on lucerne pellets is repeatable when the same animals received ryegrass-based pasture (Jonker et al., 2017, 2020).

A range of measures associated with meat yield and quality were measured. The traits are briefly described below, with full descriptions provided by Brito et al. (2017) and Payne et al. (2009).

#### Live animal

The liveweight (LWT, kg) at 8 months of age (or if missing, at 6 months of age) was extracted from the SIL database, and to ensure that animals were comparable, data were scaled to a mean liveweight of 42 kg. The preslaughter liveweight (PRESLT, kg) was recorded prior to slaughter at approximately 8 months of age. The dimensions of the M. longissimus (colloquially known as the eye muscle) were made over the 12th rib by a commercial ultrasound operator, specifically eye muscle depth (EMD, mm), width (EMW, mm), and fat depth over the muscle (FDM, mm).

#### Carcass

The carcass yield and quality traits, measured post-slaughter, varied depending on the source of the animals but included hot carcass weight (kg), butt circumference (CBUTT, cm), GR fat depth over the 12th rib at a distance of 110 mm from the midline (CGRM, mm), carcass length (CLGTH, cm), and leg length (LEGLGTH, cm). For a proportion of the animals that were processed through Alliance Group Ltd. processing plants, the traits estimated using the two-dimensional imaging system VIAscan^®^ (Mclean et al., 2006) were available. VIAscan^®^ (VS) traits included carcass weight (VSCWT, kg), GR as an estimate of carcass fat depth (VSGR, mm), and the percentage of lean meat in the carcass (VSYLD, %), leg (VSLEG, %), loin (VSLOIN, %), and shoulder (VSSHLD, %). The dressing percentage was calculated as the ratio of carcass weight divided by PRESLT (DO%).

The carcass weight (Carc Wt) is a consolidated trait, and for the majority of animals, it represents hot carcass weight; if the hot carcass weight was not measured on an animal, then the cold carcass weight or VSCWT was used, which is directly derived from the hot carcass weight.

#### Meat Quality

For a proportion of the animals, as indicated in Table 1, at 24 h post-slaughter, the carcass fat color (L*, a*, and b*, using a Minolta Chromometer) was measured on the external surface of the carcass (FATL, FATA, and FATB) as described by Payne et al. (2009), and the pH measurements were made on the M. longissimus (carcass pH; CPH). In the boning room, the area of the M. longissimus (colloquially referred to as the carcass eye muscle area, CEMA) and muscle color (L*, a*, and b*) were measured using a Minolta Chromometer, after allowing a fresh-cut surface of the muscle (colloquially referred to as the loin) to bloom for 30 min (LOINL, LOINA, and LOINB). The M. longissimus was then collected post the boning room, for frozen and tenderness measurements (shear force, SHF, kgF) that were subsequently undertaken as described by Brito et al. (2017). For the other animals, as indicated in Table 1, the M. longissimus was collected, and the measurements were undertaken at the Invermay Agricultural Centre as described by Brito et al. (2017). Specifically, the loin pH (LPH), marbling score, and muscle color and tenderness, as described earlier, were measured. The marbling score (MARB) was assessed on a 1–5 scale and used as a predictor of intramuscular fat content in the M. longissimus in lambs (Brito et al., 2017; Guy et al., 2019).

The carcass data included records from the research and CPT flocks, and data from surplus ram lambs from the high and low methane selection lines (60 born in 2012 and 60 born in 2013). The meat quality data were only available on the ones from the research and CPT flocks.

#### Statistical analyses

With the exception of the methane trait data, which were stored in an independent database, all other traits and animal data were extracted from the SIL database, together with pedigree information.

Measures of CH_4_ emissions were expressed as gross emissions (CH_4_, g/d) and methane yield (g CH_4_/kg DMI). Intake was based on the measured DMI on the day of the methane measurement. A randomized block design was used, and significant systematic effects and covariates for gross CH_4_ and CH_4_/DMI were determined using a general linear model procedure (SAS 2015).

The details of the final models used for the different traits are given in Table 1. For all traits, fixed effects fitted in initial models included birth/rearing rank (brr; born and reared as a combination of triplets, twins, and singletons: 33, 32, 31, 22, 21, and 11) and age of dam at the time of the animal’s birth (aod: 2–5 years). Birthday deviation within a year (bdev) or age at slaughter deviation within a year (SltrAgeDev) were fitted as covariates. The final contemporary groups fitted depended on the trait, but all included birth flock (flk: 2638, 3633, 4640, 4757, or 9153), birth year (byr: 2002–2013), and sex (male or female). Additionally, for the methane traits, recording year of methane measurements (ryr: 2011–2014), lot within a year (lot: mob of 96 animals, 1–5), group within a lot (group: sub-mob of up to 24 animals measured contemporaneously), and round of measurement, as each animal was measured twice with at least 2 weeks between measurements (round: 1 or 2), were also utilized to establish contemporary groups. Specifically, the contemporary groups for methane traits were byr.flk.sex (cg4) and ryr.lot.group.round (cg5). For the non-methane traits, the mob at the time of the measurements was also used to construct contemporary groups. 

The final models for each carcass trait are described in Table 1. Interactions between the fixed effects were tested together with weight traits as covariates and, by a process of backward elimination, the parsimonious models were selected.

The phenotypic, genetic, and environmental variances and covariances between methane emissions and carcass or meat quality traits were estimated using the univariate and bivariate (two-trait) analyses undertaken using ASReml 3.0 (Gilmour et al., 2009).

For ultrasound and early liveweight measures from live animals (i.e., preslaughter), all 1,825 animals that had been measured for methane also had records for these traits, therefore, a direct estimation of the genetic and phenotypic correlations was possible. For post-slaughter data, however, the numbers varied per trait. The analysis, therefore, primarily used pedigree relationships to estimate methane. This enabled the use of detailed carcass data collected from all flocks to provide estimates of genetic and phenotypic correlations with methane traits. There were some traits where there were no animals with both methane and the slaughter trait recorded. These were specialist measures of fat color, meat color, pH, and eye muscle area traits recorded on CPT animals. For these traits, the genetic and phenotypic correlations were completely extrapolated based on correlations scaled by relatedness using a numerator relationship matrix within a linear mixed model, as described in detail by Gilmour et al. (2009). Heritabilities were classified as low, if less than 0.15; as moderate, if 0.15—0.30; and as high, if greater than 0.30 (Brito et al., 2017).

## Results

The heritabilities for total methane production and methane yield were 0.25 and 0.11, respectively, while the heritabilities for carcass traits were moderate to high (0.14—0.65) (Table 2). The standard errors were low, indicating that estimates were accurate and significantly different from zero.

The gross methane production was positively genetically correlated with liveweight, carcass weight traits, and eye muscle depth and width, while negatively correlated with CGRM-a predictor of carcass fat (Table 3). These significant genetic correlations were also reflected in the phenotypic correlations except for CGRM; however, VSGR had a significant negative phenotypic correlation. The additional significant phenotypic correlations were FDM, dressing-out percentage, butt circumference, and carcass length.

**TABLE 3:** Phenotypic (r_p_) and genetic (r_g_) correlations between methane emissions (g CH_4_/d) and methane yield (g CH_4_/kg DMI/d) with live animal, carcass, and meat quality traits. Significant correlations (estimate is greater than twice the standard error) are given in bold. See Table 1 for trait descriptions.

	With CH_4_/DMI, g/kg/d			With CH_4_, g/d	
	No. Records	r_p_ (s.e.)	r_g_ (s.e.)	r_p_ (s.e.)	r_g_ (s.e.)
Live animal					
LWt	27,158	0.03 (0.02)	0.08 (0.11)	**0.54 (0.01)**∗	**0.78 (0.04)**∗
PRESLT	25,304	0.05 (0.04)	0.08 (0.13)	**0.44 (0.03)**∗	**0.56 (0.08)**∗
FDM	23,217	−0.01 (0.02)	−0.05 (0.12)	**0.10 (0.02)**∗	−0.01 (0.09)
EMD	22,339	0.01 (0.02)	0.03 (0.12)	**0.13 (0.03)**∗	**0.26 (0.09)**∗
EMW	20,195	−0.003 (0.02)	0.08 (0.12)	**0.08 (0.03)**∗	**0.27 (0.10)**∗
Carcass					
Carc Wt	26,009	0.04 (0.07)	0.002 (0.13)	**0.53 (0.04)**∗	**0.49 (0.09)**∗
DO%	23,664	−**0.16 (0.08)**	−0.12 (0.14)	−**0.06 (0.03)**∗	−0.20 (0.11)
CGRM	18,290	**0.20 (0.08)**	0.03 (0.14)	−0.03 (0.10)	−**0.24 (0.11)**∗
CLGTH	16,985	0.08 (0.07)	0.03 (0.14)	**0.24 (0.06)**∗	0.17 (0.12)
LEGLGTH	16,979	−**0.18 (0.08)**	0.02 (0.14)	0.09 (0.09)	0.12 (0.12)
CBUTT	15,219	0.08 (0.07)	−0.06 (0.15)	**0.25 (0.06)**∗	0.22 (0.12)
CEMA	15,686	0.01 (0.05)	0.04 (0.19)	0.13 (0.07)	0.25 (0.16)
VSCWT	25,552	0.04 (0.07)	0.02 (0.13)	**0.53 (0.04)**∗	**0.52 (0.09)**∗
VSGR	19,564	−0.03 (0.09)	0.03 (0.13)	−**0.24 (0.08)**∗	−0.16 (0.11)
VSLEG	25,220	−0.001 (0.08)	−0.07 (0.13)	0.09 (0.08)	0.07 (0.10)
VSLOIN	25,220	0.02 (0.08)	−0.05 (0.14)	−0.08 (0.09)	0.13 (0.11)
VSSHLD	25,220	−0.14 (0.07)	−0.15 (0.12)	−0.10 (0.08)	−0.04 (0.10)
VSYLD	25,220	−0.05 (0.08)	−0.10 (0.12)	−0.01 (0.08)	0.05 (0.10)
Meat quality					
CPH	14,743	−0.01 (0.03)	−0.08 (0.25)	−0.01 (0.04)	−0.19 (0.23)
LPH	12,905	−0.03 (0.03)	−0.17 (0.20)	0.03 (0.04)	0.01 (0.16)
FATL	14,855	0.04 (0.03)	0.23 (0.23)	−0.03 (0.04)	−0.17 (0.20)
FATA	14,854	0.001 (0.04)	0.03 (0.24)	0.09 (0.05)	0.31 (0.20)
FATB	14,855	0.004 (0.04)	0.03 (0.22)	−0.01 (0.05)	−0.07 (0.19)
LOINL	14,777	0.02 (0.03)	0.15 (0.21)	0.05 (0.04)	0.15 (0.19)
LOINA	14,775	−0.001 (0.04)	0.02 (0.25)	−0.03 (0.05)	−0.24 (0.22)
LOINB	14,770	0.001 (0.03)	0.01 (0.26)	0.03 (0.04)	−0.05 (0.24)
MARB	11,201	0.03 (0.04)	0.17 (0.19)	0.01 (0.04)	−0.15 (0.16)
SHF	12,959	0.03 (0.03)	0.15 (0.18)	0.01 (0.04)	−0.04 (0.15)

∗ Correlations significant at the 5% level (p<0.05).

There were no significant genetic correlations between methane yield and carcass and quality traits. There was a significant positive phenotypic correlation with CGRM and negative correlations with dressing-out percentage and leg length.

Although not significant, the VIAscan^®^ lean yield traits tended to have a negative (i.e., favorable) genetic correlation with methane yield.

## Discussion

As the initial progenitors of the divergent methane lines were measured in the NZ central progeny test (CPT) flocks, and methane is heritable, we can predict methane yields in CPT animals. This meant that the associated detailed carcass data collected on CPT animals could inform likely trends and effects of selecting for methane in the NZ commercial sheep population.

Heritability estimates for all carcass traits were moderate to high and close to previous estimates in New Zealand sheep (Jopson et al., 2009; Brito et al., 2017). Absolute methane emissions (g/d) were significantly and positively associated with liveweight and associated traits. This is unsurprising as the sheep were fed at a fixed feeding level 2.0 × maintenance metabolizable energy requirements in which liveweight is the main driver of the absolute feed offer (CSIRO, 1990) and with increasing body weights, the animal receives more feed which in turn leads to higher emissions (Swainson et al., 2018; Van Lingen et al., 2021). However, methane yield had no significant correlation with liveweight.

As expected, gross methane emissions were positively genetically correlated with liveweight but less so with carcass weight. There was a low negative genetic correlation between gross methane and dressing-out percentage and also a significant low negative genetic correlation with carcass fat. For methane yield, no genetic correlations with any of the meat and carcass traits were significant. But there were some significant phenotypic correlations that were similar to those previously published, such as higher fat content associated with higher methane yield and lower dressing-out percentage (Pinares-Patino et al., 2013; Elmes et al., 2014). These results together with those previously published suggest a growing body of evidence that selecting for lowered methane yield could lead to animals that are slightly leaner with a higher carcass yield per kilogram of body fat. This is in keeping with the evidence that breeding for lowered methane emissions selects for a reduced acetate to propionate ratio (Jonker et al., 2020). Propionate promotes gluconeogenesis and energy metabolized by the liver, whereas acetate is the primary source of energy metabolism in adipose tissues (Bergman, 1990). Given that the carcass meat yield and dressing-out percentage are traits of considerable economic importance in many sheep industries, it would be useful in future studies investigating selection for altered carcass composition to also monitor rumen volatile fatty acids and methane emissions in order to better estimate this genetic relationship.

## Conclusion

These results show that the use of breeding as a mitigation strategy for lower methane yields and the resulting physiological changes do not negatively affect meat quality or carcass traits. Low-emitting animals may even have greater economic value through slightly higher dressing-out rates, decreased fat, and increased meat yields. These results are of importance for all ruminant livestock production systems. They are of particular relevance for meeting the globally agreed targets through the development of robust selection indices that aim to reduce methane emissions while also increasing productivity. Meat production and meat quality are increasingly important goals in sheep production and therefore accurate estimates of genetic and phenotypic parameters of these traits with methane production are essential for any industry implementation. The current work will underpin methane reduction *via* genetics in the New Zealand industry.

## Data Availability

The original contributions presented in this study are included in the article/Supplementary Material; further inquiries can be directed to the corresponding author.
