# Supplementary material for: Can we have our steak and eat it: The impact of breeding for lowered environmental impact on yield and meat quality in sheep
Source: Front Genet. 2022 Sep 16;13:911355. doi: 10.3389/fgene.2022.911355 (PMC9523426; doi:10.3389/fgene.2022.911355)
Supplement: Supplementary file 1 [file Table1.DOCX]

**APPENDIX 1**

Methane, live animal, ultrasound, carcass and meat quality trait description with associated birth years and birth flocks each trait was recorded in.

|  | Birth Years by Birth Flock^1^ | | | | |
| --- | --- | --- | --- | --- | --- |
| Trait Description | Flock 1 | Flock 2 | Flock 3 | Flock 4 | Flock 5 |
| *Methane* |  |  |  |  |  |
| Gross methane, g/d | 2010-13 | 2009-11 | 2007, 2009-13 | 2007, 2009-11 | 2007, 2009-11 |
| Methane yield, g/kg DMI | 2010-13 | 2009-11 | 2007, 2009-13 | 2007, 2009-11 | 2007, 2009-11 |
| *Live animal* |  |  |  |  |  |
| Live weight (aged 8 months), kg | 2010-13 | 2002-13 | 2002-13 | 2003-13 | 2002-13 |
| Pre-slaughter weight, kg | 2012-13 | 2009-13 | 2002-13 | 2003-13 | 2004-13 |
| Ultrasound fat depth, mm | 2010-13 | 2002-03, 2005-13 | 2002-13 | 2003-05, 07-11 | 2002-06,09,11 |
| Ultrasound eye muscle^2^ depth, mm | 2010-13 | 2002-03, 2005-13 | 2002-13 | 2003-04, 07-11 | 2002-04, 09,11-13 |
| Ultrasound eye muscle^2^ width, mm | 2010-13 | 2003, 2005-13 | 2005-13 | 2003-04, 07-11 | 2002-04, 09,11-13 |
| *Carcass* |  |  |  |  |  |
| Carcass weight^3^, kg | 2012-13 | 2002-13 | 2002-13 | 2003-13 | 2005-13 |
| Carc Wt /PRESLT, % | 2012-13 | 2009-13 | 2002-13 | 2003-13 | 2005-13 |
| GR, mm | 2012-13 | 2002-06, 2008-13 | 2003-13 | 2012-13 | 2012-13 |
| Carcass length, cm | 2012-13 | 2002-06, 2008-12 | 2003, 2006-13 | 2012-13 | 2012-13 |
| Leg length, cm | 2012-13 | 2002-06, 2008-12 | 2003, 2006-13 | 2012-13 | 2012-13 |
| Butt circumference, cm | 2012-13 | 2002-06, 2009-13 | 2010-13 | 2011-13 | 2011-13 |
| Eye muscle area, cm^2^ |  |  | 2002-12 | 2003-06 | 2005-07 |
| VIAscan carcass weight, kg | 2012-13 | 2002-12 | 2002-13 | 2003-13 | 2005-13 |
| VIAscan GR, mm | 2012-13 | 2002-12 | 2004-13 | 2009-13 | 2009-13 |
| VIAscan leg lean yield, % | 2012-13 | 2002-12 | 2002-13 | 2003-13 | 2005-13 |
| VIAscan loin lean yield, % | 2012-13 | 2002-12 | 2002-13 | 2003-13 | 2005-13 |
| VIAscan shoulder lean yield, % | 2012-13 | 2002-12 | 2002-13 | 2003-13 | 2005-13 |
| VIAscan total lean yield, % | 2012-13 | 2002-12 | 2002-13 | 2003-13 | 2005-13 |
| *Meat Quality* |  |  |  |  |  |
| Carcass pH |  |  | 2002-13 | 2012-13 | 2012-13 |
| Loin pH |  | 2002-05, 2008-13 | 2008-12 | 2008-11 | 2008-11 |
| Carcass fat colour L* |  |  | 2002-13 | 2012-13 | 2012-13 |
| Carcass fat colour a* |  |  | 2002-13 | 2012-13 | 2012-13 |
| Carcass fat colour b* |  |  | 2002-13 | 2012-13 | 2012-13 |
| Carcass loin colour L* |  |  | 2002-13 | 2012-13 | 2012-13 |
| Carcass loin colour a* |  |  | 2002-13 | 2012-13 | 2012-13 |
| Carcass loin colour b* |  |  | 2002-13 | 2012-13 | 2012-13 |
| Marbling score^4^ |  | 2008-13 | 2009-12 | 2009-11 | 2009-11 |
| Tenderness, shear force, KgF |  | 2002-05, 2008-12 | 2008-12 | 2008-11 | 2008-12 |

^1^Flock 1: Sheep Improvement Limited (SIL) Flock 3633; Flock 2: SIL Flock 2638; Flock 3: SIL Flock 4640; Flock 4: SIL Flock 4757; Flock 5: SIL Flock 9153

^2^Eye muscle is the colloquial name for the *M. longissimus*

^3^Carcass weight represents either hot carcass weight, cold carcass weight or VIAscan® carcass weight

^4^Marbling is measured using a subjective score where 1 = no marbling through 5 = high levels of marbling
